# Mental Health of the Prison Medical Workers (PMWs) and Influencing Factors in Jiangxi, China

**DOI:** 10.3390/ijerph14121459

**Published:** 2017-11-26

**Authors:** Xiaojun Liu, Dongdong Jiang, Zhaoxun Hou, Meikun He, Yuanan Lu, Zongfu Mao

**Affiliations:** 1 School of Health Sciences, Wuhan University, 115# Donghu Road, Wuhan 430071, China; xiaojunliu@whu.edu.cn (X.L.); 2017203050046@whu.edu.cn (D.J.); houzhaoxun@whu.edu.cn (Z.H.); 2017203050026@whu.edu.cn (M.H.); 2 Global Health Institute, Wuhan University, 115# Donghu Road, Wuhan 430071, China; yuanan@hawaii.edu; 3 Department of Public Health, University of Hawaii at Mānoa, Honolulu, HI 96822, USA

**Keywords:** mental health, influencing factors, prison medical workers (PMWs), symptom checklist-90-R (SCL-90-R)

## Abstract

Prison medical workers (PMWs) are critically important, but they are also vulnerable to psychological problems. Currently, there is no study on examining PMWs’ mental health conditions and possible influencing factors in China. Hence, we conducted this cross-sectional survey, aiming to understand the mental health status of the PMWs and related impact factors in Jiangxi province of China. We employed the Chinese version of the Symptom Checklist-90-R (SCL-90-R) to assess the mental disorders and psychological health conditions of PMWs in Jiangxi. The *t* tests were used to compare the differences for the average score of SCL-90-R between the Chinese general population and targeted PMWs of this study. Multivariable logistic regression analyses were conducted to identify the main factors associated with overall detection rate of PMWs’ psychological health conditions. The scores of four dimensions (somatization, obsessive-compulsive symptoms, anxiety, and paranoid ideation) were significantly higher than the Chinese national norm, and the total positive rate was 49.09% among the PMWs. Gender, marital status, age, and length of employment are identified to be the most significant predictors to affect PMWs’ mental health. Positive correlations between each of the nine dimensions of the SCL-90-R have been verified. This study demonstrated for the first time that PMWs are facing mental health risk and suffering serious psychological problems with psychopathology symptoms, which has become a growing concern in China. Our current findings suggest a need for more in-depth studies on this subject going forward to validate our conclusions and also to identify more impact factors, since such studies and knowledge of PMWs’ mental health and influencing factors are very limited in China.

## 1. Introduction

Globally, law enforcement officials have be one of the highest-risk and highest-pressure occupations, and prison guards are more vulnerable [1,2,3]. Because of the special working environment, the prison guards are responsible for managing a group of special people (mostly criminals). Thus, prison guards have a higher working risk and more stressful mental health conditions as compared to other types of jobs. There are some empirical studies, particularly those conducted in some developed countries in Europe and America, focusing on the investigation and evaluation of the psychological health status quo and the influencing factors among this special population [2,3,4,5,6]. However, very few studies have been conducted in low- and middle-income countries, especially in China. On the other hand, there are prison medical workers (PMWs) in China who are essential, because they serve both as the health providers and guards in the prison. They provide daily primary health care or psychological counseling and psychotherapy to many prisoners, but no research has been done concerning their physical or mental health condition. Furthermore, there are no other health professionals like social workers or psychologists in China’s prisons. Therefore, PMWs in China include only the general doctors and nurses who work in prisons.

As the special kind of police groups in China, the PMWs need to assist the prison guards in carrying out the prison management. Due to the particularity of the management objectives—to provide daily primary health care or psychological counseling and psychotherapy for many prisoners, and to reduce conflict—PMWs are often faced with the potential harms or threats when they are dealing with prison breaks, suicides, fights, and other issues caused by prisoners. As the health care providers, PMWs not only provide the primary health care services to the prisoners, but also offer them some irreplaceable services such as psychological counseling, psychotherapy, and both physical and psychological health education. Therefore, PMWs are critical in reforming the criminals and protecting their lives and health. However, PMWs are usually overburdened, stressed, and under high pressure, which may result in a variety of negative effects, such as psychological problems and symptoms of psychopathology, including somatization, obsessive-compulsive symptoms, interpersonal sensitivity, depression, and anxiety.

However, the importance of psychological problems of PMWs and related factors have not been realized in China and there are a very few studies conducted to focus on the prison system workers, neither their physical fitness nor mental health. There has been no study preformed to examine PMWs’ psychological health conditions and their possible influencing factors in China. Since PMWs are preferentially vulnerable for psychological problems, in this paper, we particularly selected PMWs as our study objects. Correspondingly, the aims of this survey study are to understand the current psychological health status of the PMWs in Jiangxi and its possible influencing factors. During the study, participants were asked anonymously and independently to answer the Chinese version Symptom Checklist-90-R (SCL-90-R) questionnaires which researchers use widely to assess a range of psychological issues and symptoms of psychopathology [7,8,9,10,11].

## 2. Materials and Methods

### 2.1. Research Design and Setting

The data used in the present study were obtained from a cross-sectional survey study in Jiangxi province of China. According to *Civil Servant Law of the People’s Republic of China* [12], all the PMWs in Jiangxi province who were in-service and officially hired by the government were included in this study, and those who were currently out of work (e.g., on vacation, engaged in in-service learning, or on sick-leave) were excluded. With the permission and help of the local authorities, survey questionnaires were distributed by a correspondent in each prison, and returned back to us after all survey questionnaire were finished. Our survey was conducted anonymously and our study subjects were asked to answer the questionnaires independently.

### 2.2. Measures

In this study, we employed the Chinese version SCL-90-R to assess the mental disorders and psychological health conditions of PMWs in Jiangxi province. The SCL-90-R, edited by Derogatis in the 1976, is designed to evaluate a broad range of psychological problems and symptoms of psychopathology, and has been verified as a popular and useful tool [7,8,9,10,11]. In the mid-1980s, Chinese scholars introduced and developed a Chinese version of the SCL-90-R [13,14], which has been widely used in diagnosing or measuring the progress and outcome of psychiatric and psychological treatments or for research purposes in China [10,15,16,17]. The good internal consistency reliability (Cronbach’s alpha coefficients ranged from 0.78 to 0.96) of the Chinese version of SCL-90-R was also confirmed [16,17,18,19]. In the current study, the Cronbach’s alpha coefficient was 0.92. The Chinese version of SCL-90-R consists of nine dimensions (contains no “Others”) with 90 items. The primary symptom dimensions that are assessed are somatization (SOM), obsessive-compulsive symptoms (OCS), interpersonal sensitivity (INTS), depression (DEPR), anxiety (ANX), hostility (HOS), phobic anxiety (PHOA), paranoid ideation (PARI), and psychoticism (PSY). All questions utilized a 4- or 5-point Likert scale, and it takes approximately 10 to 15 minutes to complete the questionnaire [9,20].

### 2.3. Quality Control

Any potential bias was successfully avoided by inducing prompts of investigators since the questionnaire were filled out through anonymous quotes taken from the participants, with no mention of personal details. The questionnaires were answered by participants independently based on their true feelings about the items. More critically, with the permission and invaluable help of the local authorities, we had at least one trained investigator in each prison to hand out the questionnaires appropriately and returned back with a higher recovery rates. We also removed those questionnaires that had poor quality of responses (e.g., the answers are exactly the same in all questions). Lastly, we used EpiData version 3.1 (The EpiData Association, Odense, Denmark) to set up the database, and the data was double entered separately by two researchers into two different files and was cross-checked twice to ensure the accuracy of the data.

### 2.4. Statistical Analysis

All the data analyses for this study were performed using Statistical software the Statistical Package for the Social Sciences (SPSS) version 23.0 for Windows (SPSS Inc., Chicago, IL, USA). The *p*-value was set at <0.05 to be considered as statistically significant. Descriptive statistics frequencies and proportions were used to summarize the sample characteristics. We used *t* tests to compare the differences for the averages scores of SCL-90-R (each of the nine dimensions) between the Chinese general population and PMWs in this study. Multivariable linear regression models were performed to assess the potential influencing factors that may affect PMWs’ mental health conditions, and simultaneously, multivariable logistic regression analyses were also conducted to identify the main factors associated with overall detection rate of PMWs’ mental disorders and psychological health conditions. Furthermore, the coefficient of product–moment correlations obtained from the person correlation analysis were used to evaluate the possible association between each of the nine dimensions of the SCL-90-R.

### 2.5. Ethical Statements

This study was conducted in accordance with the Declaration of Helsinki, and the study protocol was reviewed and approved by the related local authorities (like the Provincial Prison Central Hospital of Jiangxi) and the Institutional Review Board of School of Health Science (IRB2016-S039A), Wuhan University. All participants were gave their informed consents for inclusion before they participated in the study.

## 3. Results

### 3.1. Sample Characteristics

A total of 350 questionnaires (since there are totally of approximately 500 PMWs in Jiangxi province) were distributed to different prisons in Jiangxi during the spring of 2017, and 337 questionnaires were returned. Seven questionnaires were excluded for this study due to poor quality (e.g., the answers are exactly the same in all questions) and the other 330 enrolled for data analysis, yielding an effective recovery rate of 94.29%. Among these 330 PMWs, 177 (53.64%) were female. The participants were classified into three groups basing on their ages, ≤30, 31–, 46–60 accounting for 42.73%, 47.27%, and 10.00%, respectively. The majority of participants are young and at middle age. Seventy percent of participants (231) are married or live together with their partner. The PMWs’ education attainment in China is known to be high and 79.09% of the respondents (261) in this study have at least a bachelor’s degree. About 18.18% of the PMWs have worked in prisons for at least 3 years, 40.00% have been employed between 4 to 10 years, and 41.82% have been employed 11 years or longer. The demographic information of the survey participants is summarized in Table 1.

### 3.2. Respondents’ Scores in Dimensions of SCL-90-R

Table 2 records the respondents’ scores in nine dimensions of SCL-90-R. The scores of four dimensions (SOM, OCS, ANX, and PARI) for PMWs were significantly higher than the Chinese national norm [15], indicating higher risk and more serious psychological problems and symptoms of psychopathology associated with PMWs in Jiangxi, China, especially in SOM (*t* = 2.19, *p* = 0.03), OCS (*t* = 5.49, *p* < 0.001), ANX (*t* = 2.53, *p* = 0.01), and PARI (*t* = 3.31, *p* = 0.001). We found no statistical differences between the national norm and the practical scores in INTS (*t* = −0.02, *p* = 0.98), DEPR (*t* = −0.12, *p* = 0.91), HOS (*t* = 1.29, *p* = 0.20), and PHOA (*t* = 1.81, *p* = 0.07). What is more, the good news is that the psychoticism rate among PMWs seemed better than the Chinese general population (*t* = −2.20, *p* = 0.02).

Table 3 illustrates some potential factors associated with respondents’ SOM, OCS, ANX, and PARI with standardized coefficients. PMWs’ marital status is the common influencing factor for OCS, ANX, and PARI; age is the common influencing factor for SOM, OCS, ANX, and PARI; education level only influences PMWs’ anxiety; PMWs’ length of employment is the common influencing factor for SOM, OCS, and ANX.

### 3.3. Detection Rate of SCL-90-R and Influencing Factors among the PMWs

**Figure 1:**
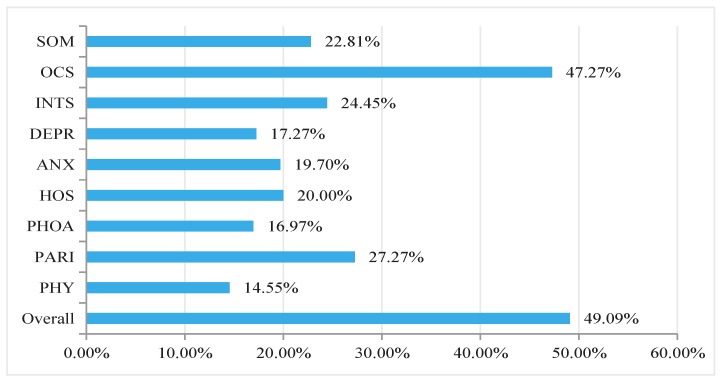
Detection rate in each dimensions of SCL-90-R among the PMWs.

According to the screening principle of the SCL-90-R, that is, scores for individual dimensions that are higher than 2 points should be considered positive and need further professional diagnosis. Figure 1 reveals that the total positive rate was 49.09% among the PMWs, and the detection rate of the top three dimensions were OCS (47.27%), PARI (27.27%), and INTS (24.45%). We also found that 22.81% responders might be exposure to somatization, 20.00% might be exposure to hostility, 19.70% might be exposure to anxiety, 17.27% might be exposure to depression, 16.97% might be exposure to phobic anxiety, 14.55% might be exposure to psychoticism (Figure 1).

In order to better identify the main factors to be associated with the overall detection rate of PMWs’ mental health issues, crude odds ratios (COR) and adjusted odds ratios (AOR) were obtained from univariate logistic regression and multivariable logistic regression, respectively. The results are presented in Table 4. Univariate logistic regression showed that female PMWs are more vulnerable to psychological health problems (COR = 2.46, 95% CI = 1.58–3.83) as compared to males. PMWs who are not married or who lived alone are more likely to suffer from mental health problems (COR = 2.81, 95% CI = 1.72–4.61). Compared to the other age groups, the middle-aged one (31–45) exhibited a more healthy condition of mental psychology (COR = 0.36, 95% CI = 0.22–0.57). PMWs with a bachelor's degree or above (COR = 0.55, 95% CI = 0.32–0.94) or a service time of 11 or longer in prisons (COR = 0.35, 95% CI = 0.18–0.65) may experience slightly psychological problems and symptoms of psychopathology. In addition, the multivariable logistic regression demonstrated that PMWs’ gender, marital status, age, and length of employment were the most significant predictors. Specifically, female PMWs are more vulnerable to psychological health problems (AOR = 2.32, 95% CI = 1.40–3.86) as compared to males. PMWs who are not married or lived alone are more likely to suffer from mental health problems (AOR = 1.48, 95% CI = 1.37–3.00). Compared to the other age groups, the middle-aged group (31–45) exhibited a more healthy psychological condition (AOR = 0.66, 95% CI = 0.33–0.94). PMWs with an 11 years or longer term of service in prisons may experience slightly higher rates of psychological problems and symptoms of psychopathology (AOR = 0.73, 95% CI = 0.28–0.86).

### 3.4. Associations between Different Dimensions of the SCL-90-R

Finally, Pearson correlation analysis was performed to evaluate the potential association between each of the nine dimensions of the SCL-90-R, and we demonstrated that all nine dimensions of the SCL-90-R were strongly correlated with each other (the minimum correlation coefficient is 0.71 and the maximum correlation coefficient is 0.96), as shown in Table 5.

## 4. Discussion

Since the SCL-90-R was introduced into China in 1984 by Wang, Z.Y. [13], it has been developed into the Chinese version of SCL-90-R and employed for a lot of related studies to investigate and explore mental health conditions among college students, primary and secondary school students, soldiers, and general populations in China [10,13,14,15,16,17]. However, very few studies have focused on special populations, such as the elders, police officers, medical workers, or specifically PMWs. Hence, this paper serves as the first study in China that aims to explore the current situations of PMWs’ mental health conditions and their potential influencing factors. This baseline information of working environment and physical and mental health conditions for PMWs is important and essential to attract more attention and support from the relevant government departments, social non-governmental organizations, and social scientists.

In total, there are around 500 PMWs in Jiangxi province, and 330 were enrolled and used for analysis in the present study. The sample demographic characteristics shown in this study are in line with the actual situation of PMWs in Jiangxi province, illustrating the good quality and high representativeness of our sample, which, therefore, means the statistical results are reliable and persuasive.

The present paper showed that the SCL-90-R score for each of the nine dimensions obtained from the PMWs was different from the Chinese national norm. Specifically, the scores for the four dimensions (SOM, OCS, ANX, and PARI) were significantly higher than those obtained from the Chinese national norm (Table 2). The Chinese national norm represents the general level of psychological health of Chinese general population. PMWs seemed to be in the worse psychological health status, and most notably somatization, obsessive-compulsive symptoms, anxiety, and paranoid ideation. Hence, the results of this study hinted that PMWs were particularly vulnerable to suffering from mental health and emotional problems. Totally, the detection rate (positive rate) was 49.09%, and OCS, PARI, and INTS were the top three symptoms of psychopathology among the PMWs in Jiangxi (Figure 1). These results suggested that the psychological issues and symptoms of psychopathology among the PMWs in Jiangxi are under a worrying circumstance, indicating that more humanistic care and social support is needed to protect this special population.

Our study also examined the possible risk factors associated with PMWs’ psychological health and revealed that PMWs’ marital status and age are related to OCS, ANX, and PARI, and PMWs with SOM, OCS, and ANX are associated with their working length. In addition, there are differences in anxiety at different educational levels among the PMWs (Table 3). Adjusted odds ratios exposed that the female and single PMWs are particularly vulnerable to the psychological problems. This observation may be mainly due to there being less social support and financial security since, currently, females in China earn much less than men on average as well as having fewer promotion opportunities. In addition, females in China are largely expected to manage the household and take care of the elderly and the children at home. Single PMWs may feel lonely and have no people to talk to at home. Some of the PMWs were interviewed when we obtained these results, one of the interesting findings associated with young PMWs is that, some young staff even started to worry about finding a spouse if they do not change their PMW job. Adjusted odds ratios also confirmed that the age group of 31–45 PMWs with 11 service years or more are in good mental health condition, which may be mainly due to their stronger social adaptability and ability to gain more community social supports with more effective and accessible social resources.

Finally, the significant and strong associations between the nine individual dimensions of the SCL-90-R are proven again, and the correlation coefficients ranged from 0.71 to 0.96 in the current study (Table 5). These results are consistent with the conclusions reported by previous studies [15,18,19,21,22,23,24].

## 5. Limitations

In the present cross-sectional survey study, we could not explore how these psychological problems or symptoms of psychopathology are interacted with each other. For example, whether or not somatization will translate into obsessive-compulsive symptoms, or in the opposite path. Moreover, some existing studies have shown that health related quality of life and supports from the social network and community may cause a series of psychological problems such as despondence, anxiety, and sadness [25,26,27,28]. However, these areas were not investigated in this study. Hence, further studies are needed for more in-depth explorations on these aspects with more scientific, rational, and rigorous design, such as cohort studies or case-control studies with Social Support Rating Scale or other survey tools, to properly addressed these conundrums

## 6. Conclusions

This study has clearly shown that the scores of four dimensions (SOM, OCS, ANX, and PARI) were significantly higher than the Chinese national norm. The total positive rate was 49.09% among the PMWs, which means that the PMWs are at higher risk and have more serious psychological problems and symptoms of psychopathology. The PMWs’ gender, marital status, age, and length of employment are the most significant predictors for their mental health. Our study has verified obviously positive correlations between each of the nine dimensions of the SCL-90-R. Hence, we suggested that humanistic care and social support are needed for this special occupational group. Moreover, the relevant government departments—especially the public security, judicial administrations, and police departments—need to give PMWs more provide more financial support and address their spiritual needs.

This study will become a starting point to draw the public’s attention to PMWs, including their physical fitness, mental health, and working conditions. The present paper presents these authentic and valuable findings, which form the baseline information about PMWs’ mental condition to get more attentions and support from the local government and the public. In addition, findings from this study have formed a basis for more studies in the future to validate our findings and to identify more risk factors concenring PMWs’ mental health.

## Figures and Tables

**Table 1:** Demographic information of the survey participants (*n* = 330).

Demographics	Frequency	Percentage (%)
**Gender**		
Male	153	46.36
Female	177	53.64
**Marital status**		
Married/live together	231	70.00
Single (spinsterhood, divorced, widowed)	99	30.00
**Age**		
≤30	141	42.73
31–45	156	47.27
46–60	33	10.00
**Education level**		
<Bachelor’s degree	69	20.91
≥Bachelor’s degree	261	79.09
**Length of employment**		
≤3 years	60	18.18
4–10 yeas	132	40.00
≥11 years	138	41.82

**Table 2:** Respondents’ scores in dimensions of SCL-90-R (*n* = 330).

Dimensions	Norm ($\bar{x}\pm s$)	Practical Score ($\bar{x}\pm s$)	*t*	*p*
**SOM**	1.48 ± 0.54	1.54 ± 0.53	2.19	0.03
**OCS**	1.83 ± 0.64	2.04 ± 0.69	5.49	0.000
**INTS**	1.68 ± 0.65	1.68 ± 0.71	−0.02	0.98
**DEPR**	1.70 ± 0.65	1.70 ± 0.72	−0.12	0.91
**ANX**	1.55 ± 0.55	1.63 ± 0.59	2.53	0.01
**HOS**	1.64 ± 0.63	1.69 ± 0.72	1.29	0.20
**PHOA**	1.40 ± 0.50	1.46 ± 0.58	1.81	0.07
**PARI**	1.58 ± 0.63	1.69 ± 0.62	3.31	0.001
**PSY**	1.53 ± 0.56	1.47 ± 0.51	−2.20	0.02

Note: scores are presented as mean ± standard deviation ($\bar{x}\pm s$).

**Table 3:** Possible factors associated with respondents’ SOM, OCS, ANX, and PARI (Standardized Coefficients).

Variables	SOM		OCS		ANX		PARI	
	Beta	*p*	Beta	*p*	Beta	*p*	Beta	*p*
Gender	−0.05	0.37	0.01	0.88	−0.08	0.21	−0.09	0.11
Marital status	−0.06	0.44	0.23	0.00	0.18	0.02	0.12	0.13
Age	0.23	0.01	0.34	0.000	0.24	0.01	0.24	0.01
Education level	0.12	0.06	−0.11	0.06	−0.13	0.04	−0.01	0.83
Length of employment	−0.17	0.04	−0.45	0.000	−0.18	0.03	−0.13	0.13

**Table 4:** Factors that are associated with overall detection rate of participants’ mental health issue.

Demographics	Negative	Positive	Crude OR (95%CI)	Adjusted OR (95%CI)
	*n* = 168 (50.91%)	*n* = 162 (49.09%)		
Gender				
Male	96 (62.75)	57 (37.25)	-	-
Female	72 (40.68)	105 (59.32)	2.46 (1.58–3.83) ***	2.32 (1.40–3.86) ***
Marital status				
Married/living together	135 (58.44)	96 (41.56)	-	-
Single	33 (33.33)	66 (66.67)	2.81 (1.72–4.61) ***	1.48 (1.37–3.00) **
Age				
≤30	53 (37.59)	88 (62.41)	-	-
31–45	98 (62.82)	58 (37.18)	0.36 (0.22–0.57) ***	0.66 (0.33–0.94) **
46~60	17 (51.52)	16 (48.48)	0.57 (0.26–1.22)	1.48 (0.48–4.59)
Education level				
<Bachelor’s degree	27 (39.13)	42 (60.87)	-	-
≥Bachelor’s degree	141 (54.02)	120 (45.98)	0.55 (0.32–0.94) *	0.87 (0.45–1.67)
Length of employment				
≤3 years	21 (35.00)	39 (65.00)	-	-
4–10 yeas	63 (47.73)	69 (52.27)	0.59 (0.31–1.11)	1.16 (0.55–2.41)
≥11 years	84 (60.87)	54 (39.13)	0.35 (0.18–0.65) ***	0.73 (0.28–0.86) *

Note: * *p*-value < 0.05; ** *p*-value < 0.01; *** *p*-value < 0.001.

**Table 5:** Correlations between respondents’ scores in individual dimensions of SCL-90-R.

Dimensions	SOM	OCS	INTS	DEPR	ANX	HOS	PHOA	PARI	PSY
**SOM**	1	-	-	-	-	-	-	-	-
**OCS**	0.77 **	1	-	-	-	-	-	-	-
**INTS**	0.80 **	0.79 **	1	-	-	-	-	-	-
**DEPR**	0.78 **	0.91 **	0.89 **	1	-	-	-	-	-
**ANX**	0.79 **	0.89 **	0.90 **	0.95 **	1	-	-	-	-
**HOS**	0.71 **	0.90 **	0.86 **	0.96 **	0.93 **	1	-	-	-
**PHOA**	0.80 **	0.78 **	0.88 **	0.80 **	0.83 **	0.80 **	1	-	-
**PARI**	0.89 **	0.75 **	0.89 **	0.83 **	0.85 **	0.78 **	0.82 **	1	-
**PSY**	0.82 **	0.80 **	0.87 **	0.92 **	0.89 **	0.87 **	0.81 **	0.86 **	1

Note: ** Correlation is significant at the 0.01 level (2-tailed).
